# Intereye structure–function relationship using photopic negative response in patients with glaucoma or glaucoma suspect

**DOI:** 10.1038/s41598-022-17868-8

**Published:** 2022-08-16

**Authors:** Jihye Lee, Seong Ah Kim, Jiyun Lee, Chan Kee Park, Kyoung In Jung

**Affiliations:** Department of Ophthalmology, Seoul St. Mary’s Hospital, College of Medicine, The Catholic University of Korea, 222 Banpo-daero, Seocho-ku, Seoul, 137-701 Korea

**Keywords:** Diseases, Medical research

## Abstract

We evaluated the intereye structure–function relationship in glaucoma patients using photopic negative response in electroretinogram analysis. Patients with confirmed glaucoma (36 eyes, 36 patients) or suspected glaucoma (19 eyes, 19 patients) were included in this study. Electroretinogram (RETI-scan) was performed with red stimulus on blue background. Intereye comparison for 55 patients was performed between better eyes and worse eyes, which were divided based on average retinal nerve fiber layer (RNFL) thickness measured using spectral-domain optical coherence tomography. In the intereye analysis, PhNR amplitude was lower in worse eyes than in better eyes (*P* < 0.001). The intereye difference in PhNR amplitude was significantly correlated with intereye difference in average RNFL, as well as average or minimum ganglion cell-inner plexiform layer (GCIPL) thickness (*P* = 0.006, 0.044, 0.001). In patients with mean deviation ≥ − 6 dB of worse eyes, the intereye difference in PhNR amplitude was significantly associated with intereye difference in average RNFL thickness or minimum GCIPL thickness (*P* = 0.037, 0.007), but significant correlation was not found between mean sensitivity of visual field tests and structural parameters. In conclusion, PhNR performed well with regard to intereye structure–function association in glaucoma patients, especially at the early stage.

## Introduction

Glaucoma is characterized by progressive loss of retinal ganglion cells (RGCs) and corresponding visual field (VF) defects. Standard automated perimetry (SAP) is commonly performed to evaluate visual function in glaucoma patients. VF tests are intrinsically subjective and affected by learning effects, fatigue, and cognitive skills of patients^1,2^. Electrophysiological tests are considered as supplementary and relatively objective functional tests compared to perimetry, even though they have not been appreciated as an essential diagnostic tool in glaucoma clinics to date^3^.

Regarding electrophysiological tests, accumulating clinical evidence suggests that pattern electroretinogram (ERG) or photopic negative response (PhNR) of the ERG has relative efficacy for glaucoma assessment^4^. Previously, our group reported that pattern ERG N95 amplitude showed better diagnostic ability for glaucoma than did SAP 24–2 in pre-perimetric glaucoma patients^5^. Pattern ERG requires an appropriate fixation and refraction similar to perimetry^2^. PhNR is a negative wave that appears following b-wave in photopic full-field ERGs^6^ and was found to be lower in patients with glaucoma^6–8^. PhNR is relatively independent of refractive errors, fixation control, and clear optics compared to pattern ERG^2,9^.

Organ pairs generally show symmetricity even though they are not invariably identical^10,11^. As clinical diseases develop and progress, asymmetry between organ pairs can exist^10,11^. Glaucoma could be bilateral or unilateral^12^. In the early stage of glaucoma, intereye asymmetry is frequent^12^. Intereye structural asymmetry in cup-to-disc ratio or peripapillary retinal nerve fiber layer (RNFL) thickness and macular ganglion cell-inner plexiform layer (GCIPL) thickness has been found to be an early sign of glaucomatous damage^11,13,14^. For functional tests, SAP has a limitation in early detection of glaucoma because substantial (approximately 25–35%) loss of RGCs is needed to detect abnormalities via SAP^15^. To detect early functional change in glaucoma patients, more sensitive functional tests are needed. In eyes with suspected or early glaucoma, a growing body of evidence has reported that PhNR amplitude was sensitive to detect the disease and had a significant association with peripapillary RNFL thickness^7,16,17^. There is a possibility that intereye difference in PhNR amplitude could reflect structural asymmetry appropriately in early glaucoma patients. Intereye difference could be independent of systemic factors such as age, systemic vascular disease, or environmental factors. Previously, the intereye difference in PhNR amplitudes was reported to be positively related to the intereye difference in relative afferent pupillary defects in asymmetric optic nerve disorders, some of which included glaucoma^18^. However, no study has evaluated the intereye structure–function relationship between PhNR amplitude and structural parameters associated with glaucoma.

Therefore, we investigated interindividual and intereye structure–function relationship for PhNR amplitudes in patients with glaucoma or suspected glaucoma. Specifically, the PhNR amplitudes were compared between better and worse eyes divided based on peripapillary RNFL thickness in patients with glaucoma or suspected glaucoma. We also analyzed the relationship between intereye differences of PhNR amplitudes and intereye differences of structural optical coherence tomography (OCT) parameters according to glaucoma stage.

## Methods

This cross-sectional study was approved by the Institutional Review Board of The Catholic University of Korea, Seoul, Korea, and was performed according to the tenets of the Declaration of Helsinki. Patients with diagnosis of glaucoma in at least one eye (the fellow eye should be diagnosed as glaucoma or glaucoma suspect) who met the inclusion criteria were enrolled at the glaucoma clinic of Seoul St. Mary’s Hospital between December 2016 and June 2017. Inclusion criteria were a best-corrected visual acuity ≥ 20/30, open angle, and axial length less than 29 mm. Patients with current or history of uveitis, brain disease that could affect vision, or vision-threatening retinal diseases such as retinal vein obstruction, macular degeneration, macular edema, or retinal detachment were excluded. Patients with far advanced glaucoma with a mean deviation (MD) less than − 20 dB were excluded. Patients with a history of intraocular surgery within six months were also excluded. Informed consent was achieved from all patients.

### Interindividual & intereye analyses

Eyes with glaucomatous optic disc such as diffuse or focal rim loss, notching, and RNFL defects with corresponding VF damage were diagnosed as having glaucoma. Eyes with glaucomatous structural damage without VF damage were diagnosed as glaucoma suspect. When both eyes satisfied the inclusion criteria for glaucoma, the worse eye was selected for interindividual analysis. For intereye analysis, better and worse eyes were divided based on peripapillary RNFL thickness evaluated by spectral domain OCT. Eyes with identical peripapillary RNFL thickness in both eyes were excluded from intereye analysis. Subgroup intereye analysis according to glaucoma stage was based on a mean deviation (MD) of − 6 dB.

### Measurements

All participants received a full ophthalmic examination, including slit-lamp examination, Goldmann applanation tonometry, central corneal thickness and axial length measurements, gonioscopic evaluation, red-free fundus photography, and stereoscopic optic disc photography.

#### Optical coherence tomography

With Cirrus SD-OCT version 6.0 (Carl Zeiss Meditec, Inc.), peripapillary RNFL thickness was measured using the optic Disc Cube 200 × 200 scan mode, and GCIPL thicknesses was determined through GCA software using a macular cube scan. The protocol by which peripapillary RNFL and GCIPL thicknesses were assessed was previously described in detail^19,20^. Average peripapillary RNFL thickness and average and minimum GCIPL thickness (thinnest GCIPL thickness over a single meridian crossing the annulus) were used in this study. Only images with signal length > 6 and without misalignment or segmentation error were included.

#### Visual field testing

All patients underwent a SAP 24–2 test with a Humphrey field analyzer (Carl Zeiss Meditec, Dublin, CA), using the Swedish interactive threshold algorithm standard strategy. Glaucomatous VF defects were defined as a cluster of three or more points having lower sensitivities less than 5% of the normal population on the pattern deviation plot. One of the abnormal points should have lower sensitivity less than 1% of the normal population. MD and pattern standard deviation (PSD) were analyzed. Reliable examinations were considered as those with less than 20% fixation losses, false positives, or false negatives.

#### Full-field photopic ERG

**Figure 1:**
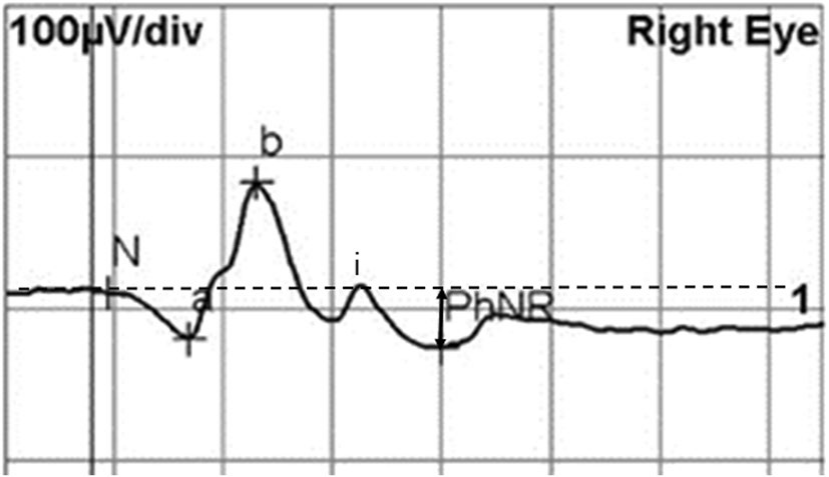
Representative electroretinogram by red stimulus on blue background. a-wave, b-wave, i-wave, and photopic negative response (PhNR) were indicated. The PhNR amplitude was determined from baseline to the negative trough of the negative peak at ~ 80 ms after b-wave and i-wave.

ERG was recorded in binocular mode with full-field LED stimulator (RETI-port Roland Consult, Germany). Patients were equipped with Dawson-Trick-Litzkow electrodes on the cornea, with reference electrodes in the forehead, and ground electrodes on the lateral canthi. Pupils were dilated with fixed combinations of phenylephrine/tropicamide (Mydrin P). Patients were light adapted for 10 min before recording. PhNR was recorded by red stimuli of 1.6 cd s/m^2^ on a blue background of 25 cd/m^2^ corresponding to the International Society for the Clinical Electrophysiology (ISECV) extended protocol^21^. The stimulus was delivered at 1.089 Hz with a duration of 4 ms. Signals were amplified with a band-pass filter of 1–300 Hz. The phNR was determined as the negative-going wave, which appears following the b-wave. The a-wave amplitude was assessed from baseline to the first negative trough, while the b-wave amplitude was measured from the trough to the peak of first positive wave. The PhNR amplitude was measured from baseline to the negative trough of the negative peak at ~ 80 ms after b-wave and i-wave (Fig. 1). The i-wave was designated as the first positive wave after the b-wave^22^. The PhNR/b-wave amplitude ratios were analyzed.

### Statistical analysis

The program SPSS for Windows version 23.0 (SPSS Inc., Chicago, IL, USA) was adopted for all statistical analysis. For interindividual comparison, Student’s t-test was used for continuous variables and the chi-square test for categorical variables. For intereye comparison, paired t-test was performed. To evaluate the relationships between structural and functional parameters, linear (*y* = a*x* + b) and logarithmic [y = log(x) + b]) regression analyses were conducted. The goodness-of-fit for the regression models was determined as the coefficient of determination, R^2^. *P* < 0.05 was regarded to indicate statistical significance.

## Results

### Interindividual analysis

Nineteen patients (eyes) with glaucoma suspect and 36 patients (eyes) with glaucoma were enrolled in this study. There was no significant difference between age, sex, corneal thickness, spherical equivalent, or axial length between groups (all *P* > 0.05, Table 1). The number of glaucoma medication was higher in the glaucoma group than the glaucoma suspect group (*P* = 0.007). MD was lower and PSD was higher in the glaucoma group compared to the glaucoma suspect group (both *P* < 0.001).

**Table 1:** Demographics of patients with glaucoma or suspected glaucoma.

	Glauocma suspect (n = 19 eyes, 19 patients)	Glaucoma (n = 36 eyes, 36 patients)	*P* value
Age (years)	50.94 ± 12.06	51.19 ± 12.74	0.945
Male/Female	5/14 (26.3%/73.7%)	15/21 (41.7%/58.3%)	0.378
Central corneal thickness (µm)	548.74 ± 38.18	528.17 ± 52.79	0.141
Spherical equivalent (diopter)	− 2.08 ± 3.44	− 2.24 ± 2.62	0.848
Axial length (mm)	24.18 ± 1.57	24.93 ± 1.46	0.084
Intraocular pressure (mmHg)	15.3 ± 3.1	13.8 ± 3.3	0.109
Number of glaucoma medication (n)	1.2 ± 1.0	2.0 ± 0.9	**0.007**
SAP 24–2 MD (dB)	− 0.99 ± 1.40	− 5.82 ± 4.64	** < 0.001**
SAP 24–2 PSD (dB)	1.58 ± 0.47	6.85 ± 4.05	** < 0.001**

MD, mean deviation; PSD, pattern standard deviation; SAP, standard automated perimetry.

*Statistically significant differences between two groups (*P* < 0.05) by student’s t-test for continuous variables or chi-squared test for categorical data are indicated in bold.

Average RNFL as well as average and minimum GCIPL thickness were lower in the glaucoma group than in the glaucoma suspect group (all *P* < 0.001, Table 2). Among ERG parameters, only PhNR amplitude was decreased in the glaucoma group (29.08 ± 10.42 µV) compared to the glaucoma suspect group (35.94 ± 10.79 µV, *P* = 0.026).

**Table 2:** Electroretinograms, retinal nerve fiber layer (RNFL) thickness, and ganglion cell-inner plexiform layer (GCIPL) thickness in patients with glaucoma or suspected glaucoma.

	Parameters	Glauocma suspect (n = 19 eyes, 19 patients)	Glaucoma (n = 36 eyes, 36 patients)	*P* value (better vs worse)
ERG	a wave amplitude (µV)	32.57 ± 10.13	29.60 ± 7.89	0.235
	b wave amplitude (µV)	112.38 ± 19.50	102.44 ± 26.85	0.160
	PhNR amplitude (µV)	35.94 ± 10.79	29.08 ± 10.42	**0.026**
	PhNR/b wave ratio	0.32 ± 0.08	0.29 ± 0.11	0.294
Spectral domain OCT	Average RNFL thickness (µm)	90.89 ± 8.83	69.36 ± 9.17	** < 0.001**
	Average GCIPL thickness (µm)	81.63 ± 4.23	67.69 ± 8.99	** < 0.001**
	Minimum GCIPL thickness (µm)	77.89 ± 4.98	56.00 ± 13.59	** < 0.001**

ERG, electroretinogram; GCIPL, Ganglion cell-inner plexiform layer; OCT, optical coherence tomography; RNFL, retinal nerve fiber layer.

*Statistically significant differences between two groups (*P* < 0.05) by student's t-test are indicated in bold.

Average RNFL in addition to average and minimum GCIPL thickness were linearly and logarithmically associated to PhNR amplitude or mean sensitivity of SAP (all *P* < 0.005, Table 3).

**Table 3:** The relationship between mean retinal nerve fiber layer (RNFL) or ganglion cell-inner plexiform layer (GCIPL) thickness and photopic negative response (PhNR) or mean sensitivity of standard automated perimetry in patients with glaucoma or suspected glaucoma.

Spectral domain OCT		PhNR amplitude (ERG)				Mean sensitivity (visual field)			
		Linear		Logarithmic		Linear		Logarithmic	
		r^2^	*P* value	r^2^	*P* value	r^2^	*P* value	r^2^	*P* value
RNFL thickness	Average	0.235	** < 0.001**	0.183	**0.001**	0.237	** < 0.001**	0.205	**0.001**
GCIPL thickness	Average	0.117	**0.011**	0.089	**0.027**	0.201	**0.001**	0.176	**0.001**
	Minmum	0.155	**0.003**	0.112	**0.013**	0.307	** < 0.001**	0.255	** < 0.001**

ERG, electroretinogram; GCIPL, Ganglion cell-inner plexiform layer; OCT, optical coherence tomography; PhNR, photopic negative response; RNFL, retinal nerve fiber layer.

Statistically significant values (*P* < 0.05) are in bold.

The PhNR amplitude was linearly and logarithmically related to mean sensitivity of SAP (r^2^ = 0.108, *P* < 0.001; r^2^ = 0.106, *P* = 0.001, respectively).

### Intereye analysis

Among a total of 55 patients, two who showed the same average RNFL thickness in the right and left eyes were excluded from intereye analysis. The number of the glaucoma medication was higher in the worse eyes than the better eyes (*P* = 0.020, Table 4). Worse eyes showed lower MD and higher PSD than better eyes (both *P* < 0.001).

**Table 4:** Demographics of patients with glaucoma: Intereye comparison.

	Better eye (n = 53)	Worse eye (n = 53)	P value
Age (years)	50.8 ± 12.5		
Male/Female	17/36 (32.1%/67.9%)		
Central corneal thickness (µm)	538.4 ± 48.1	535.5 ± 51.4	0.068
Spherical equivalent (diopter)	− 1.8 ± 2.7	− 1.8 ± 3.0	0.619
Axial length (mm)	24.6 ± 1.5	24.7 ± 1.5	0.103
Intraocular pressure (mmHg)	14.2 ± 3.1	14.3 ± 3.1	0.718
Number of glaucoma medication (n)	1.6 ± 1.1	2.0 ± 0.9	**0.020**
SAP 24–2 MD (dB)	− 2.2 ± 2.2	− 4.4 ± 4.1	** < 0.001**
SAP 24–2 PSD (dB)	3.3 ± 2.5	5.8 ± 3.7	** < 0.001**

MD, mean deviation; PSD, pattern standard deviation; SAP, standard automated perimetry.

*Statistically significant differences between two groups (*P* < 0.05) by paired t-test are indicated in bold.

In the comparison of average RNFL and average and minimum GCIPL thickness, worse eyes exhibited decreased values than better eyes (all *P* < 0.001, Table 5). Among ERG parameters, PhNR amplitude and PhNR amplitude/b wave ratio were decreased in worse eyes compared to better eyes (*P* < 0.001).

**Table 5:** Electroretinograms, retinal nerve fiber layer (RNFL) thickness, and ganglion cell-inner plexiform layer (GCIPL) thickness in patients with glaucoma: Intereye comparison.

	Parameters	Better eye	Worse eye	P value
ERG	a wave amplitude (µV)	31.4 ± 9.0	31.1 ± 7.2	0.520
	b wave amplitude (µV)	108.2 ± 22.4	108.4 ± 20.9	0.921
	PhNR amplitude (µV)	33.7 ± 10.7	30.2 ± 9.3	** < 0.001**
	PhNR/b wave ratio	0.32 ± 0.10	0.28 ± 0.09	** < 0.001**
Spectral domain OCT	Average RNFL thickness (µm)	82.3 ± 10.9	72.7 ± 9.8	** < 0.001**
	Average GCIPL thickness (µm)	75.7 ± 7.4	70.3 ± 9.0	** < 0.001**
	Minimum GCIPL thickness (µm)	66.8 ± 13.7	58.1 ± 13.7	** < 0.001**

ERG, electroretinogram; GCIPL, Ganglion cell-inner plexiform layer; OCT, optical coherence tomography; PhNR, photopic negative response; RNFL, retinal nerve fiber layer.

*Statistically significant differences between two groups (*P* < 0.05) by paired t-test are indicated in bold.

**Figure 2:**
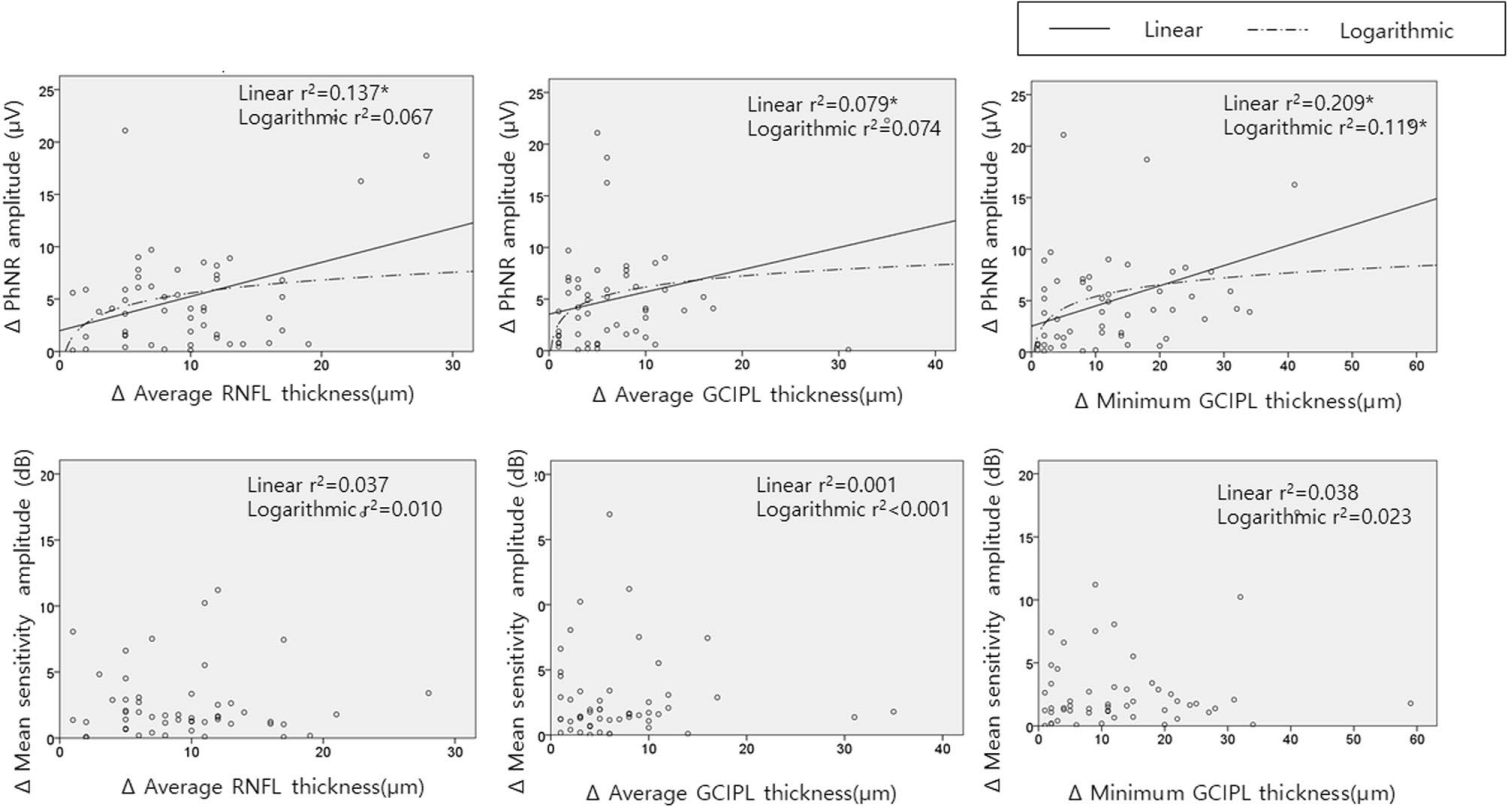
Scatter plots of interocular differences between photopic negative response (PhNR) and retinal nerve fiber layer (RNFL) thickness or ganglion cell-inner plexiform layer (GCIPL) thickness. *Regression analysis with *P* < 0.05.

The intereye difference in PhNR amplitude was linearly correlated with the intereye difference in average RNFL or average or minimum GCIPL thickness (*P* = 0.006, 0.044, 0.001, Table 6, Fig. 2). The intereye difference in mean sensitivity of the visual field test did not have a significant association, linearly or logarithmically, with the intereye difference in average RNFL or average or minimum GCIPL thickness (all *P* > 0.05).

**Table 6:** The relationship between ΔRNFL or ΔGCIPL thickness and Δ photopic negative response (PhNR) amplitude or Δ mean sensitivity of standard automated perimetry between better and worse eyes.

SD-OCT		Δ PhNR amplitude (ERG)				Δ Mean sensitivity (visual field)			
		Linear		Logarithmic		Linear		Logarithmic	
		r^2^	*P* value	r^2^	*P* value	r^2^	*P* value	r^2^	*P* value
Δ RNFL thickness	Average	0.137	**0.006**	0.067	0.061	0.037	0.170	0.010	0.476
Δ GCIPL thickness	Average	0.079	**0.044**	0.074	0.050	0.001	0.851	< 0.001	0.915
	Minmum	0.209	**0.001**	0.119	**0.012**	0.038	0.160	0.023	0.274

GCIPL, Ganglion cell-inner plexiform layer; PhNR, photopic negative response; RNFL, retinal nerve fiber layer.

Statistically significant values (*P* < 0.05) are in bold.

In patients with MD of worse eyes ≥ − 6 dB, the intereye difference in PhNR amplitude was significantly associated with the intereye difference in average RNFL thickness and minimum GCIPL thickness (*P* = 0.037, 0.009, respectively, Table 7). No correlation was found between mean sensitivity of SAP and structural parameters (all *P* > 0.05). In patients with MD of worse eyes < − 6 dB, the intereye difference between average RNFL thickness and minimum GCIPL thickness was linearly and logarithmically related to the intereye difference in PhNR amplitude or mean sensitivity of SAP (All *P* < 0.05).

**Table 7:** The relationship between ΔRNFL or ΔGCIPL thickness and Δ photopic negative response (PhNR) or Δ mean sensitivity of standard automated perimetry between better and worse eyes according to glaucoma stage.

SD-OCT		Δ PhNR amplitude (ERG)				Δ Mean sensitivity (visual field)			
		Linear		Logarithmic		Linear		Logarithmic	
		r^2^	*P* value	r^2^	*P* value	r^2^	*P* value	r^2^	*P* value
**Worse MD ≥ -6 dB**									
Δ RNFL thickness	Average	0.107	**0.037**	0.059	0.127	0.005	0.658	0.004	0.683
Δ GCIPL thickness	Average	0.076	0.085	0.056	0.143	0.002	0.789	0.003	0.752
	Minmum	0.170	**0.007**	0.079	0.075	0.007	0.596	0.015	0.451
**Worse MD < -6 dB**									
Δ RNFL thickness	Average	0.348	**0.043**	0.132	0.246	0.399	**0.028**	0.127	0.256
Δ GCIPL thickness	Average	0.114	0.284	0.200	0.145	0.018	0.681	< 0.001	0.988
	Minmum	0.495	**0.011**	0.456	0.016	0.499	**0.010**	0.283	0.075

ERG, electroretinogram; GCIPL, Ganglion cell-inner plexiform layer; MD, mean deviation; PhNR, photopic negative response; RNFL, retinal nerve fiber layer.

Statistically significant values (*P* < 0.05) are in bold.

The intereye difference in PhNR amplitude was logarithmically associated to the intereye difference in mean sensitivity of SAP (r^2^ = 0.101, *P* = 0.038), but not linearly related to that (r^2^ = 0.025, *P* = 0.244).

**Figure 3:**
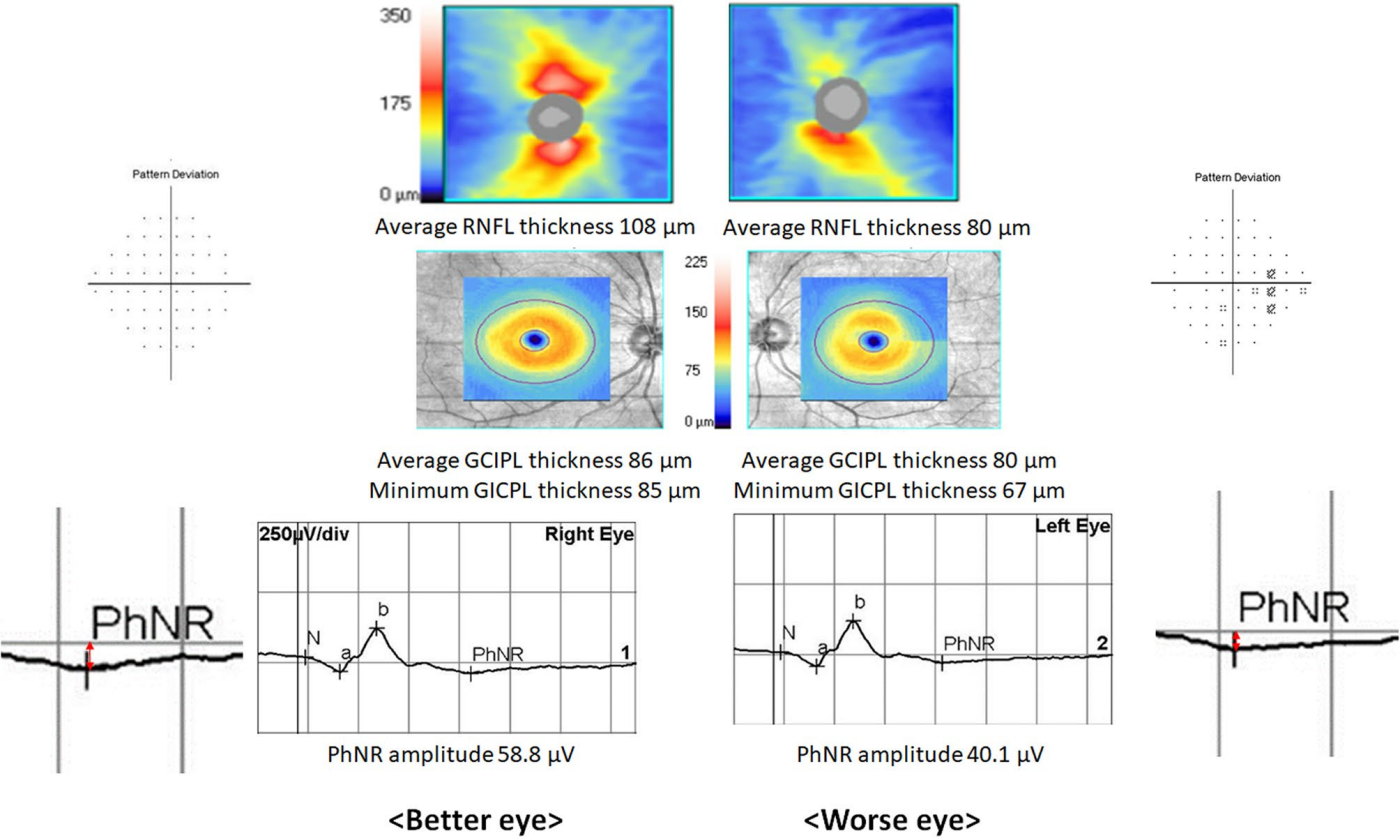
Representative case. A 44-year-old patient had thinner retinal nerve fiber layer and ganglion cell-inner plexiform layer in his left eye (80 µm, 67 µm) compared to his right eye (86 µm, 85 µm). PhNR amplitude was decreased in his left eye (40.1 µV) compared to his right eye (58.8 µV).

A representative case was shown in Fig. 3. A 44-year-old patient revealed thinner RNFL and GCIPL thickness in his left eye (80 µm, 67 µm) than his right eye (86 µm, 85 µm). The PhNR amplitude was lower in his left eye (40.1 µV) than his right eye (58.8 µV).

## Discussion

We demonstrated that eyes with glaucoma displayed lower PhNR amplitude than those with suspected glaucoma (*P* = 0.026). The RNFL or GCIPL thickness was significantly correlated to PhNR amplitude in inter-individual analysis (all *P* < 0.0.05). In intereye analysis, worse eyes exhibited lower PhNR amplitude than better eyes (*P* < 0.001). The intereye difference in PhNR amplitude had relevance to the intereye difference in OCT parameters in total eyes or in the subgroups divided according to glaucoma stage (All *P* < 0.05). However, the intereye difference in mean sensitivity was significantly related to the intereye difference in RNFL or GCIPL thickness in moderate to advanced stages of glaucoma (All *P* < 0.05), but not in early-stage glaucoma (All *P* > 0.05). The PhNR amplitude performed well in assessing intereye structure–function relationship in eyes with relatively early-stage glaucoma.

For interindividual analysis, PhNR amplitude was reduced in patients with glaucoma compared to those with suspected glaucoma. It has been reported that patients with suspected glaucoma or ocular hypertension exhibited decreased PhNR amplitude compared to normal control individuals^7,23^. Preiser et al. found that PhNR amplitude was reduced in pre-perimetric glaucoma and more so in glaucoma with VF defects, corresponding to the results of this study^24^, even though it is controversial whether there is a significant difference in PhNR amplitudes according to glaucoma stage^7,16^. We found a significant relationship between PhNR amplitude and average RNFL thickness as well as minimum or average GCIPL thickness in patients with suspected glaucoma or glaucoma. Kirkiewicz’s study demonstrated that PhNR amplitude was significantly correlated to macular nerve fiber layer thickness and the ganglion cell complex in eyes with suspected glaucoma^7^. Given those findings, PhNR amplitude seemed to decrease proportionately with glaucomatous damage in eyes with suspected glaucoma and glaucoma.

For intereye analysis, worse eyes divided based on average RNFL thickness showed attenuated PhNR amplitudes than better eyes. Okuno et al. reported that the relative PhNR amplitudes (affected/normal eye) were significantly and positively associated with the relative afferent pupillary defect in eyes with asymmetrical optic nerve damages, corresponding to our study to some degree^18^.

In normal eyes, the intereye difference in RNFL thickness is small^10^. Glaucoma is often asymmetric at the initial stage and in its deterioration, although it is frequently bilateral^10^. Intereye structural asymmetry in cup-to-disc ratio or peripapillary RNFL or GCIPL thickness could be useful in early diagnosis of glaucoma^11,13,14^. In general, OCT measures the residual thickness of RNFL and compares them to the normative database. The intereye difference in RNFL thickness might detect “loss” of RNFL caused by glaucomatous damage, especially in early-stage glaucoma^14^. In normal human samples, retinal ganglion cell numbers range from 700,000 to 2 million^25^. Measurements of absolute RNFL thickness in each eye could have a limitation in detection of glaucoma, because the range of the normative RNFL database, or the 5th and 95th percentiles of RNFL thickness, is considerably wide. Comparing right and left eyes for RNFL thickness could reflect glaucomatous structural loss independent of age or systemic conditions.

The relative difference in PhNR amplitude of worse eyes compared to better eyes might indicate glaucomatous functional “loss.” The differences in PhNR amplitudes were linearly related to the differences in OCT structural parameters. In a subgroup analysis of eyes with a MD ≥ − 6 dB, the intereye difference in RNFL or GCIPL thickness was only associated with intereye difference in PhNR amplitudes not with intereye differences in mean sensitivity of SAP. Among eyes with MD < − 6 dB, significant structure–function relationships were shown both with intereye difference of PhNR and mean sensitivity. Previously, Feuer et al. reported intereye difference in VF tests as an early sign of glaucomatous damage but did not evaluate intereye structure–function relationship. In this study, intereye difference in RNFL or GCIPL thickness parameters had a relevance to intereye difference in mean sensitivity in moderate- to advanced-stage glaucoma but not in early-stage glaucoma. SAP might not detect subtle intereye differences in RNFL or GCIPL thickness in the early stage of glaucoma, probably because a substantial (approximately 25–35%) loss of RGCs is needed to detect abnormalities in SAP^15^. A growing body of evidence found attenuation of PhNR amplitudes in eyes with ocular hypertension and suspected glaucoma^7,23^. One study reported that the PhNR amplitude was improved following IOP decreases in eyes with glaucoma and ocular hypertension, suggesting that PhNR amplitude indicates the degree of RGC function and RGC loss^26^. Early functional detection of glaucoma with PhNR amplitude probably seemed to result in a favorable intereye structure–function relationship with PhNR amplitude in early-stage glaucoma patients.

In this study, red-on-blue stimulus was employed according to the ISCEV extended protocol for PhNR^21^, because it is more sensitive and specific than white-on-white stimulus or blue-on-yellow stimulus^27,28^. Recently, PhNR has been recognized as having two classifications: PhNR1 as the trough after the b-wave and before i-wave and PhNR2 as the trough after the i-wave. To date, PhNR2 is traditionally regarded as the PhNR commonly used, and red-on-blue stimulus is appropriate for measuring PhNR2^28^. We adapted PhNR2 with red-on-blue stimulus in this study.

One of the limitations in this study is inclusion of a small number of participants. To the best of our knowledge, we performed the first study that investigated intereye structure–function relationship with PhNR. Intereye comparison has a strength because it is independent of individual variation such as age or systemic vascular diseases, which could affect glaucomatous damage. However, intereye comparison has a limitation in advanced stage of glaucoma when present in both eyes. In this study, the average MD of worse eyes was − 4.4 dB. The results from our study were confined to patients with asymmetric glaucoma without bilateral advanced glaucoma. Although PhNR performed favorably in structure–function relationship, the amplitude of PhNR form in full-field photopic ERG might be limited to visualization of focal RGC dysfunction. Further research is needed to investigate if multifocal PhNR could be used combined with full field PhNR or independently in detection of presence or progression of glaucoma^29^.

In conclusion, intereye structure–function relationship was favorably assessed with PhNR amplitude in patients with suspected glaucoma or relatively early-stage glaucoma. PhNR evaluation might be employed as a supplementary tool in the assessment of visual function when RNFL loss is not evident in SAP. Further studies are required to evaluate the diagnostic ability of intereye differences of PhNR for early detection of glaucoma and in prediction of glaucoma progression.

## Data Availability

All data generated or analysed during this study are included in this published article.
